# Outcomes of Clubfoot Treated With Casting in Ghana

**DOI:** 10.7759/cureus.14046

**Published:** 2021-03-22

**Authors:** Aditya Yadavalli, William Hennrikus, Scott Reichenbach

**Affiliations:** 1 Anesthesiology, Penn State College of Medicine, Hershey, USA; 2 Orthopaedics, Penn State Health Milton S. Hershey Medical Center, Hershey, USA; 3 Epidemiology and Public Health, Hope Walks, Mechanicsburg, USA

**Keywords:** clubfoot, ghana, africa, global health, orthopedics, public health, pediatrics

## Abstract

Objective: Idiopathic clubfoot deformity is a condition in pediatric orthopedics with a prevalence of 1 in 1000. This study reports the outcomes of clubfoot treatment in Ghana.

Methods: The study was Institutional Review Board (IRB) approved. Patients with clubfoot were treated by the Ponseti method including weekly casting, Achilles tendon lengthening (TAL), and prolonged bracing. Data points collected included: extent of clubfoot, age, relapse, tenotomy prevalence, and number of casts.

Results: Out of 1,634 patients, 72.4% were less than a year of age at the time of the first cast, 82.6% had more than eight casts prior to bracing, and 74.0% had a percutaneous Achilles tenotomy prior to the final cast placement. Only 1.2% of patients suffered a relapse.

Conclusion: In Ghana, delays in seeking in treatment are common. Optimal results for the Ponseti treatment occur in children who present prior to the age of one. In the current study, 27.6% of children delayed treatment until after one. We recommend a community advocacy program to educate leaders and medical personnel about the Ponseti method. Despite a delay of treatment in 25% of the patients, there was only a 1.2% relapse rate. We recommend the Ponseti method in Ghana for children of all ages.

## Introduction

Idiopathic clubfoot deformity is a common condition in pediatric orthopedics with a prevalence of about 1.2 in 1000 [1]. Many regions of the developing world are not able to treat clubfoot deformity due to a variety of barriers to care including a lack of resources, distance to travel, and misunderstanding of disease [1].

The inadequate surgical care of children in developing countries has not been without consequences. In many of these countries, congenital anomalies go unrepaired [1]. The consequences of pediatric surgical conditions may be life-long since they affect children at critical times during development [1]. Previous studies concerning the musculoskeletal disease burden in sub-Saharan Africa have shown that over 50% of these surgical conditions can be improved with policy changes and education reform [2]. Clubfoot is a deformity that occurs in approximately 1.2 in every 1000 live births in the world [1,3]. If the deformity is not casted or treated appropriately within the first few years of the child’s life, there could be serious consequences [4-7]. For example, untreated clubfoot is associated with difficulty walking, pain, persistent deformity, calluses, chronic infections, and societal ostracizing [8]. The goal of treatment of clubfoot is a normal functioning, pain-free, plantigrade foot with no need for supplemental casting or shoe modifications [9].

Clubfoot can be diagnosed based on visual inspection as soon as the child is born. Clubfoot presents with four clinical components-CAVE: cavus-a high arch, adductus-a turned in forefoot, varus-a turned in heel, and equinus-a plantarflexed position due to a tight heel cord [10]. Radiographs or advanced imaging are not needed. The current treatment of choice is the Ponseti method of serial casting, an Achilles tenotomy, and bracing for about three years. Recent studies prove that the rapid international acceptance of this method has been paramount in increasing the success rate in the treatment of idiopathic clubfoot [11]. Previous studies have shown that in developing countries, such as Ghana, medical resources are scarce, amplifying the importance of a rapid international implementation of the Ponseti method [12-15]. The purpose of the current study is to report on the CURE Ponseti treatment program, tenotomy rate, and relapse rate of clubfoot in Ghana, Africa. The CURE registry includes all patients treated with clubfoot in the year 2015 and collected at six different CURE sponsored sites in various regions of Ghana.

## Materials and methods

This study was approved by the Penn State College of Medicine Institutional Review Board (IRB). A retrospective chart review was conducted on a consecutive set of patients with clubfoot in Ghana treated and recorded on the CURE registry during 2015. The patient population studied included patients under the age of five years diagnosed with idiopathic congenital talipes equinovarus. A team of Ghanaians, including physicians, therapists, and technicians conducted the casting and tenotomy. After this process, patients were placed in a locally made Steenbeek brace [16]. Data points collected included presence of clubfoot, age, gender, number of casts, tenotomy prevalence, and relapse rate. Statistical analysis including basic chi-squared was performed to evaluate correlations between the parameters and post-treatment outcomes.

## Results

**Figure 1:**
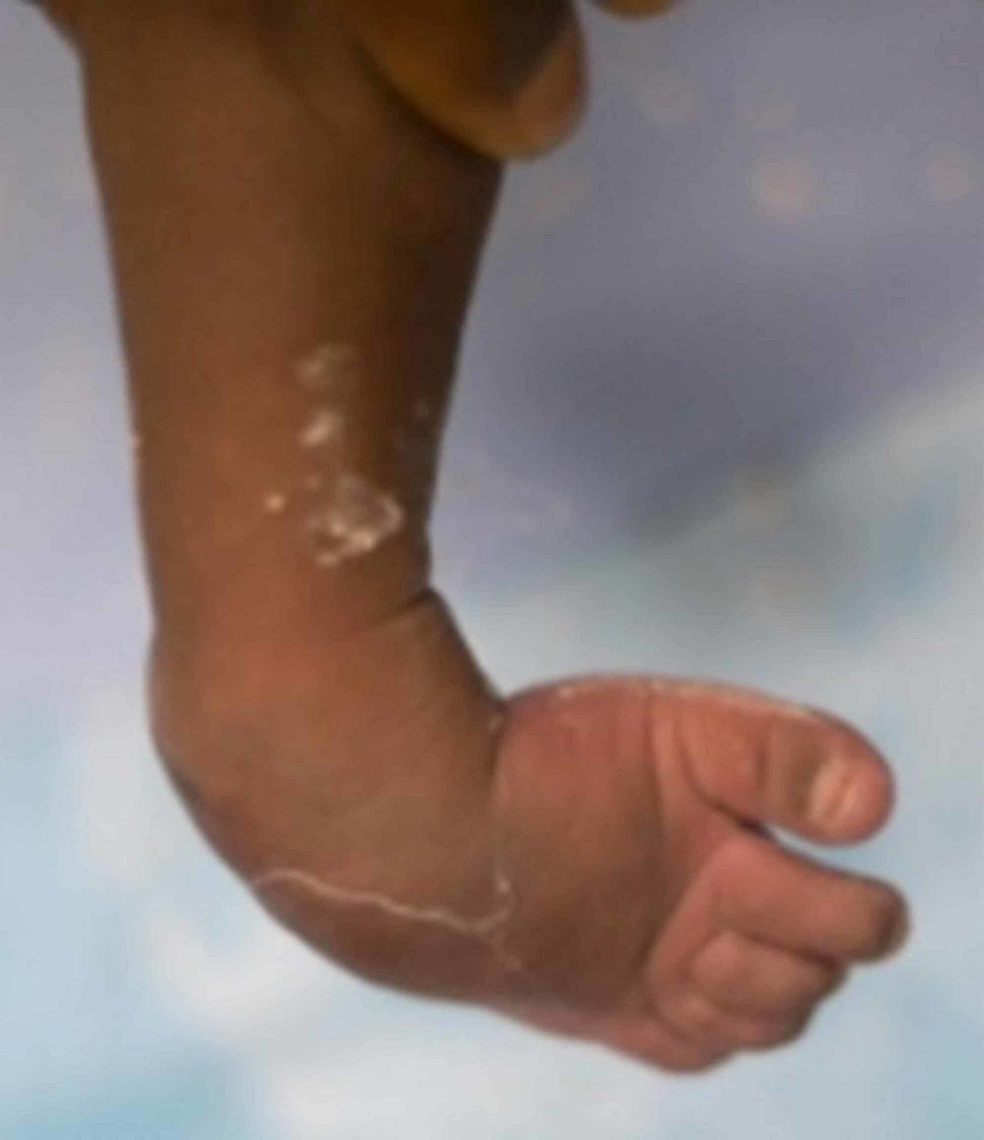
Clubfoot cavus presentation

**Figure 2:**
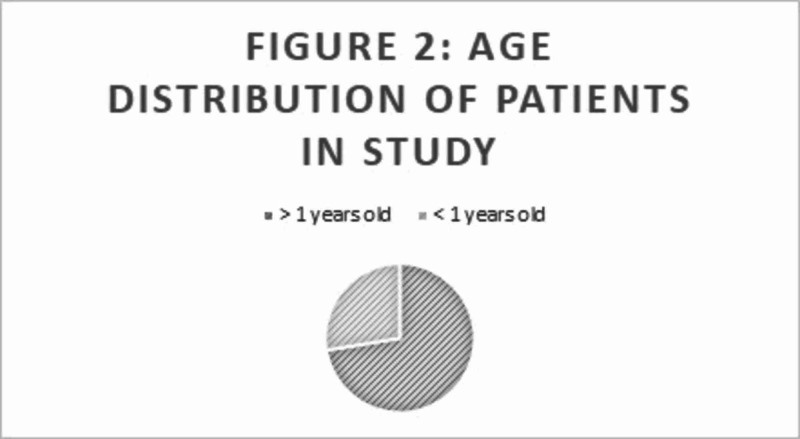
Age distribution of patients in the study

**Figure 3:**
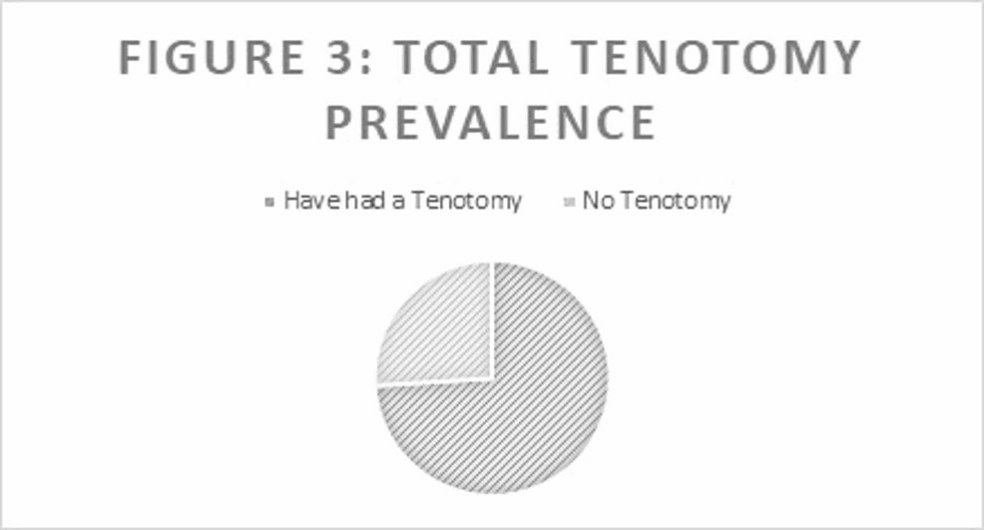
Total tenotomy prevalence

Clubfoot can be diagnosed based on visual inspection as soon as the child is born (Figure 1). A total of 1,634 patients (including 615 males and 1,019 females) were treated during the year 2015. In 2015, 872,000 births occurred in Ghana. Because all of the newborns in Ghana with clubfoot were not included in the CURE registry, the exact prevalence of clubfoot in Ghana during 2015 could not be determined. In the CURE registry, 72.4% of patients were younger than one year, and 27.6% of patients were older than one year at age of treatment (Figure 2). Also, 16.2% had less than eight casts and 82.6% had greater than eight cases prior to tenotomy and bracing; 74% had a percutaneous, Achilles tenotomy prior to the final cast placement (Figure 3). Twenty-six percent of feet were supple and corrected without the need for a tenotomy. There was a range of ages at which treatment was initiated from newborn to five years old. Finally, 1.2% of patients returned within one year due to a relapse in clubfoot following a complete Ponseti treatment protocol.

## Discussion

This study aims to report on the CURE Ponseti treatment program, tenotomy rate, and relapse rate of clubfoot in Ghana. By identifying these key characteristics, physicians in low-income countries can better customize their treatment of clubfoot for each individual patient.

The traditional teaching is that the Ponseti method effectiveness is best if initiated under the age of one [17]; 27.6% of patients in the current study were older than one at the time of treatment. This large number of patients over the age of one stresses the importance of creating advocacy programs to educate community members, leadership, and medical personnel to destigmatize the condition and emphasize early referral for the Ponseti method. As a child gets older without treatment, the probability decreases that there will be a complete correction of the deformity [17].

The gold standard treatment is the Ponseti method [18]. The cavus, adductus, and varus deformities are corrected with serial casting. Lastly, the hindfoot equinus is corrected by performing a percutaneous Achilles’ tendon release to allow the ankle to be positioned at the appropriate right angle with the leg [18].

About 20% of our patients did not require a tenotomy, indicating these patients had less extensive clubfoot. The literature suggests that the typical patient with clubfoot requires about eight casts prior to tenotomy [19]. In the current study, 82% had more than eight casts, suggesting that these patients had more extensively involved clubfoot. By comparison, Banskota reported that > 50% of Nepalese children ages one to five required eight or more casts [20]. The late presentation of clubfoot in the current study further highlights the need to improve advocacy programs and emphasize early referral for treatment. In both the current study and the Banskota study [19], late presentation up to the age of five could be successfully treated with the Ponseti method. However, cases presenting after age one often required greater than eight casts. This further lends credence to the importance of referral regardless of age. Organizations such as Hope Walks and MiracleFeet are leading efforts to educate medical providers in low-income countries such as Ghana about the efficacy of the Ponseti method in clubfoot [21,22]. Advocacy programs should highlight that late treatment can still be effective.

The current study has a few limitations including only a one-year data collection period, the lack of a control group, the inability to verify the use of the post-casting orthosis, the inability to capture all clubfoot in Ghana due to the inaccessibility of remote villages, the loss of some subjects to follow up, and the inability to calculate an exact prevalence of clubfoot in Ghana.

## Conclusions

In 2015, the Ponseti method was applied to 1634 patients in Ghana via the CURE registry. A plantigrade foot was achieved in 98% of cases. In low-income countries such as Ghana, this research will impact orthopedic healthcare providers’ knowledge on the importance of Ponseti treatment for clubfoot.
